# Isokinetic Muscle Strength and Knee Function in Anatomical Anterior Cruciate Ligament Reconstruction With Hamstring Autografts: A Prospective Randomized Comparative Study Between Suspensory and Expandable Femoral Fixation in Male Patients

**DOI:** 10.7759/cureus.32482

**Published:** 2022-12-13

**Authors:** Andreas Panagopoulos, Vasileios Giannatos, Giorgos Moros, Dimitrios Mylonas, Antonis Kouzelis, John Gliatis

**Affiliations:** 1 Orthopaedic Department, School of Medicine, University of Patras, Patras, GRC; 2 Physiotherapy Department, School of Medicine, University of Patras, Patras, GRC

**Keywords:** isokinetic muscle strength, cortical button, expandable, femoral fixation, anterior cruciate ligament (acl)

## Abstract

Background

Clinical performance, anterior knee stability, and isokinetic strength after anterior cruciate ligament (ACL) reconstruction with hamstring autografts are mainly influenced by graft selection, femoral tunnel preparation, and type of femoral fixation. Expandable femoral fixation devices are expected to provide a stronger initial fixation with circular graft compression, a blind-ended tunnel in the femur with less enlargement, and a theoretical double-band ACL equivalent through graft rotation. This study aimed to evaluate isokinetic strength and functional capacity after ACL reconstruction with hamstring tendons using two different anatomical femoral fixation techniques (expandable vs fixed-looped button).

Methodology

A total of 48 male patients with ACL deficient knees were randomized to two different femoral fixation groups, namely, the expandable (AperFix) and the standard cortical (Button) group. The primary outcome measures were isokinetic hamstrings and quadriceps strength capabilities and the hamstrings/quadriceps ratio at 60 degrees/second (°/s) and 180°/s using a Cybex before and at three, six, nine, 12, and 24 months after surgery. Secondary measurements were anteroposterior knee stability at two years (using KT-1000 arthrometer) and the functional outcome using the International Knee Documentation Committee (IKDC 2000) form, the Tegner activity scale, and the Lysholm knee score. Data were compared using a paired t-test and analysis of variance, with a p < 0.05 level of significance.

Results

Most patients regained the 60°/s quadriceps strength between three and 12 months (62.5% for the Button group vs. 50% for the AperFix group), as well as the 180°/s strength (79.17% vs 70.83%); however, at the 24-month evaluation, seven (29.17%) patients in the Button group and five (20.83%) in the AperFix group had significant deficits. The 60°/s flexor strength was regained in the first six months in 19 (79.17%) patients in the Button group and in 16 (66.7%) patients in the AperFix group, whereas the percentages for the 180°/s strength were 79.17% and 75%, respectively. Beyond the 24-month evaluation, only three (12.5%) patients in the Button group and four (16.67%) in the AperFix group had significant flexor deficits. Regarding the H/Q ratio, at 60°/s, the mean recovery time was six and 7.5 months for the Button and AperFix groups, respectively, whereas 15 and 12 patients, respectively, did not recover during the two-year duration. At 180°/s, a mean recovery time of six months was needed for the button group, and nine patients did not recover two years later. For the AperFix group, nine months were needed, and 12 patients did not recover in two years. Clinical performance and anterior knee stability showed no statistically significant differences between groups.

Conclusions

Although there were no significant differences in clinical performance, knee stability, and isokinetic strength testing between expandable and cortical button femoral fixation groups, return to play was doubtful at two years postoperatively.

## Introduction

Clinical performance, anterior knee stability, and isokinetic strength after anterior cruciate ligament (ACL) reconstruction with hamstring autografts are mainly influenced by graft selection, femoral tunnel preparation, and the type of femoral fixation (fixed-loop or adjustable button, aperture or intratunnel stabilization). However, the ideal method remains an ongoing source of debate in the literature [1-4]. Regarding femoral fixation, several systematic reviews and meta-analyses have demonstrated either similar results regarding anteroposterior knee stability, clinical outcomes, and graft failures, or better overall results with buttons, screws, pins, or other intratunnel fixation methods [5-7]. The expandable femoral devices provide homogenous circular compression of the graft inside the femoral tunnel ensuring a strong initial fixation and are characterized by the theoretical double-band effect in a single tunnel as the quadrupled hamstring graft is twisted during the insertion of the femoral device [8]. The existing literature for expandable devices is inconsistent with either similar or superior clinical outcomes, especially when the anteromedial portal is utilized [9-12].

Considering this evidence, we designed a prospective, randomized, comparative study between expandable and cortical button fixation in ACL deficient male patients using strict exclusion criteria. We found no difference in anteroposterior knee stability, tunnel enlargement (using computed tomography postoperatively and at one year), as well as significant differences in clinical scores and athletic performance at the 24-month follow-up [13]. Here, we present the results of isokinetic strength deficits at 60 degrees/second (°/s) and 180°/s, as well as the timing to return to play (RTP), assuming that the expandable fixation would provide better results compared to traditional button fixation.

## Materials and methods

Study patients

Between 2016 and 2019, 70 male patients with ACL deficiency were enrolled in the study. Informed consent and institutional review board approval (approval number: 15245/20.07.2016-77/15.04.2016) were obtained before initiating the study. Patients were equally assigned to cortical Button (n = 35) or expandable (AperFix) (n = 35) femoral fixation group using MiniTab software version 16.2.4 (MiniTab, State College, PA). A standard arthroscopic ACL reconstruction was applied through the anteromedial portal using a quadruple-bundle semitendinosus-gracilis autograft. Tibial fixation was accomplished in all patients using a sheath-screw fixation system.

Inclusion and exclusion criteria

Inclusion criteria were male patients, aged between 15 and 45 years, with an ACL injury for >three weeks. Exclusion criteria included multi-ligament knee injuries, meniscus suturing, severe cartilage defects or advanced knee osteoarthritis, severe preoperative stiffness, muscle deficits or previous knee injuries, insufficient graft diameter (<7.5 mm), and injury to the other knee. In total, 22 patients were excluded after initiation. Of these, seven missed two or more isokinetic strength tests, six had meniscus suturing despite normal preoperative magnetic resonance imaging, four had cartilage work, three received a synthetic graft due to insufficient diameter, one left voluntarily before completing the study, and another suffered a severe knee injury one month postoperatively because of a traffic accident, leaving a total of 48 patients (24 in each group) for the final evaluation.

Primary outcomes

Isokinetic quadriceps and hamstring strength were assessed for both legs at 60° and 180° using a Cybex Norm 770 dynamometer. The hamstrings to quadriceps ratio (H/Q ratio) was calculated for the above isokinetic velocities. Three submaximal and one maximal concentric repetition were executed. The non-operated leg was tested first each time. Prior to Cybex testing, all patients performed a warm-up process including 10 minutes on a stationary bike and stretching exercises. After the warm-up, each patient was positioned on the Cybex in a sitting position at a chair-back angle of 85°, with the knees at 90° and the torso and thigh secured with straps. Gravity corrections and an axial check were performed to ensure the proper position for testing. Patients were allowed to execute a few repetitions to familiarize themselves with the process. Additionally, they were given verbal explanations for the proper execution of the movements. Minimal pain was allowed during the process, especially in the third-month evaluation. After the evaluation, patients walked on a treadmill for two to three minutes, followed by static stretching allowing relaxation, and then applying ice for eight to 10 minutes. There was no injury caused by the evaluation process. The isokinetic assessment was performed preoperatively and at three, six, 12, and 24 months after the surgery.

Secondary outcomes

Patient demographic data, athletic performance, current health, type of injury, and knee status were collected preoperatively using the International Knee Documentation Committee (IKDC) forms. Functional knee status was assessed at three, six, 12, and 24 months postoperatively using the IKDC score, Lysholm score, and Tegner activity scale. Anteroposterior knee stability was evaluated at the same postoperative intervals using the KT-1000 instrument (MEDmetric Corp, San Diego, CA, USA).

Surgical technique, medication, and rehabilitation

All patients underwent inpatient ACL reconstruction under general anesthesia using a quadruple-bundle semitendinosus-gracilis graft. Three doses of second-generation cephalosporin were used for chemoprophylaxis, and low-molecular-weight heparin was prescribed for three weeks for thromboprophylaxis. As a standard practice, all grafts were washed off with hydrogen peroxide and soaked in vancomycin solution. Tendon retrieval was accomplished prior to arthroscopy without using the tourniquet, which was inflated to 350 mmHg during the arthroscopic procedure. An accessory medial portal (after marking the anatomical ACL footprint with a spine needle) was used in all patients for femoral tunnel preparation at 120° of knee flexion. In the Button group, femoral fixation was performed in a standard fashion using fixed-loop cortical buttons (from Stryker, Biomet, or DePuy companies). The length of the cradle was 25 mm in 18 cases and 20 mm in six cases. In the AperFix group, the femoral tunnel had a length of 24 mm to match the standard length of the device (Cayenne Medical, Inc, Scottsdale, Arizona), whereas the diameter of the implant (9, 10, or 11 mm) was determined by the diameter of the final graft (≤7.5, 7.5-9, or 9-10, respectively). After securing the AperFix implant through the medial portal, the tendons were passed into the knee joint in a retroverted manner to be finally retrieved via the tibia tunnel. After calibration of the graft’s tension trough, 10-15 cycles of flexion-extension, the graft was fixed to the tibia in both groups with the AperFix® II Tibial Implant (Cayenne Medical, Inc, Scottsdale, Arizona). The graft was fixed in 10-15°of flexion, and an additional staple was used in all cases. Surgical incisions were infiltrated with 30 mL of 0.25% bupivacaine, and drainage was applied for 24 hours. Patients were discharged wearing a hinged knee brace for six weeks, cold therapy instructions, and painkillers on demand. Postoperative rehabilitation was focused on active full extension, quadriceps isometric exercises, and passive kinesiotherapy. Straight-ahead running was initiated at three months, cutting or pivoting at six months, and contact sporting activities at 9-12 months postoperatively. Data were collected and recorded at three, six, 12, and 24 months.

Statistical analyses

The Shapiro-Wilk test was used initially for normalized quantitative variables. For descriptive statistics, the variables were described with mean ± standard deviation (SD). For inferential statistics, to determine associations between Button or AperFix group and each variable, we used the Student’s t-test (parametric scenario) or the Wilcoxon’s rank-sum test (non-parametric scenario). The two population proportions were compared using the binomial z-test. Finally, quantitative variables were correlated with Spearman rho, and qualitative variables were correlated with Pearson’s chi-square test. Statistical tests were considered two-sided, and statistical significance was considered at p-values of <0.05. The statistical analysis was performed using the R software for statistical computing, with the assistance of the RStudio interface (both open-source products).

## Results

All patients were evaluated at least 24 months post-surgery. The mean follow-up period was 29 ± 7.5 months. There were no demographic differences between groups (Table 1). The mean hamstring graft diameter was 8.75 ± 0.68 for the Button group and 7.98 ± 0.57 for the AperFix group (p < 0.001). The quadriceps and hamstrings deficits between the injured and uninjured leg were calculated (as a percentage) for the Button and AperFix groups preoperatively and at three, six, 12, and 24 months postoperatively. In total, 46 patients had a final isokinetic strength testing beyond 24 months (mean: 8.5 months after the 24th-month evaluation). The H/Q ratio was also calculated as it is widely used to assess readiness to return to sport and the risk of injury. The Tegner, IKDC, and Lysholm scores and the KT-1000 measurements were used as secondary measurements in this study to compare the functional status between the two groups. During data processing, as it was proven that the limb that was injured (right or left) did not alter the results, it has not been considered a confounding factor.

**Table 1:** Demographic data of the two groups. BMI = body mass index; MFC = medial femoral condyle; LFC = lateral femoral condyle

Demographic data	Button (n = 24)	AperFix (n = 24)	P-value
Age (years), mean ± SD	27 ± 7.5	28 ± 8.3	0.763
Height (cm), mean ± SD	1.77 ± 0.07	1.79 ± 0.2	0.310
Weight (kg), mean ± SD	78.46 ± 11.23	81.34 ± 13.33	0.752
BMI, mean ± SD	25.02 ± 3.64	25.24 ± 2.25	0.821
Surgery time, mean ± SD	68.33 ± 4.92	75.42 ± 4.98	<0.001
Meniscal pathology			
Normal, n (%), yes	14 (58.3%)	15 (62.5%)	0.764
Medial tear, n (%), yes	4 (16.7%)	6 (25%)	0.478
Lateral tear, n (%), yes	3 (12.5%)	0 (0%)	0.073
Medial and lateral tear, n (%), yes	0 (0%)	1 (4.2%)	0.313
Posterior horn, n (%), yes	3 (12.5%)	2 (8.3%)	0.638
Cartilage pathology			
None, n (%), yes	20 (83.3%)	20 (83.3%)	0.1
Patella, n (%), yes	1 (4.2%)	0 (0%)	0.313
MFC, n (%), yes	2 (8.3%)	4 (16.7%)	0.384
LFC, n (%), yes	1 (4.2%)	0 (0%)	0.313

Extensor and flexor strength deficits

Muscle deficits of <10% between injured and normal knees are considered normal according to the literature, and the threshold of 20% deficit has been used for RTP during isokinetic hamstrings and quadriceps testing [14].

**Figure 1:**
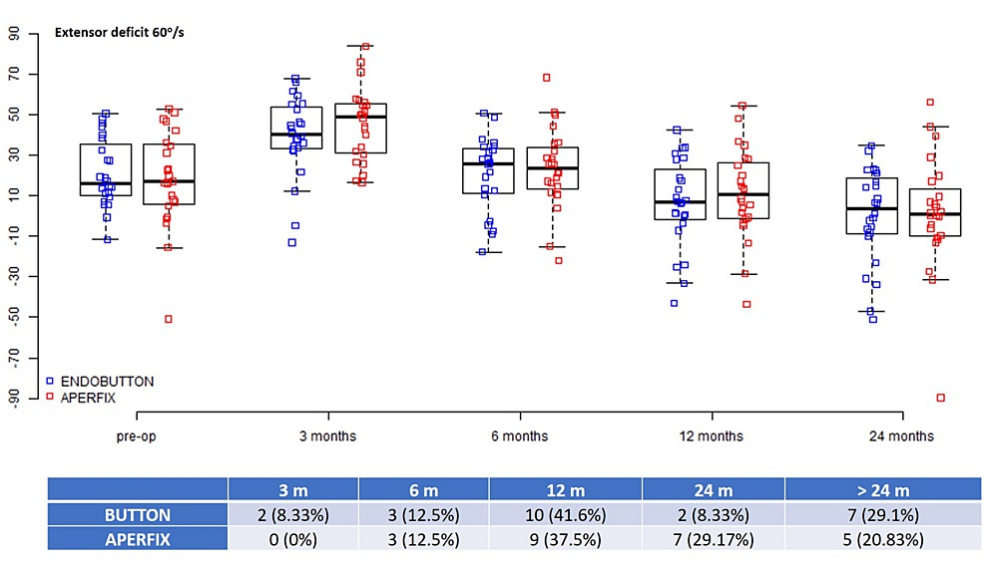
Extensor deficits at 60°/s during different time intervals.

**Figure 2:**
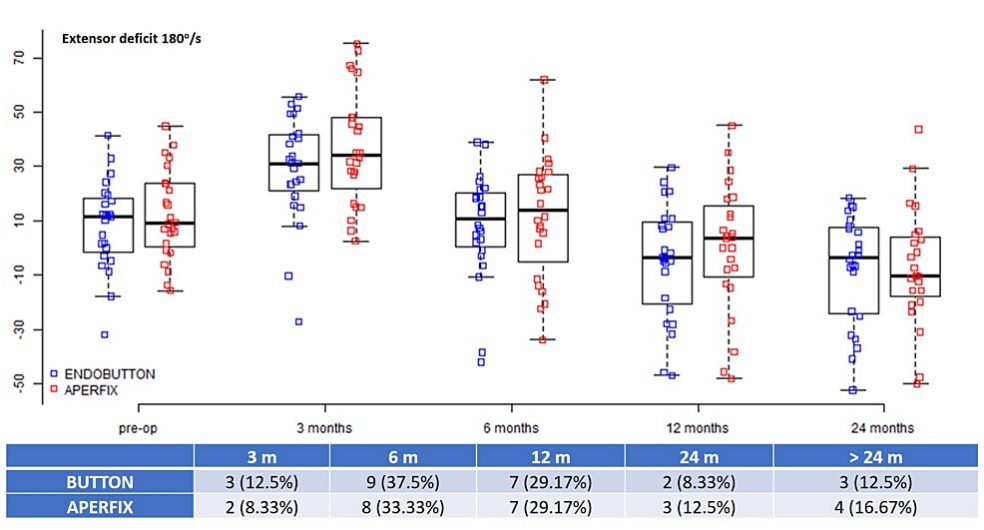
Extensor deficits at 180°/s during different time intervals.

The mean preoperative 60°/s extensor deficit was 15.93 (10.66-33.97) for the Button group and 16.79 (6.41-35.08) for the AperFix group (p = 0.862), and the 180°/s values were 8.57 ± 16.23 and 12.2 ± 16.47, respectively (p = 0.446) (Table 2). Most patients regained the 60°/s quadriceps strength between three and 12 months (62.5% for the Button group vs. 50% for the AperFix group) as well as the 180°/s strength (79.17% vs. 70.83%); however, after the 24-month evaluation, seven (29.17%) patients in the Button group and five (20.83%) patients in the AperFix group had significant 60°/s extensor deficits. Similarly, the 180°/s extensor deficit beyond 24 months was present in three (12.5%) patients in the Button group and four (16.67%) patients in the AperFix group (Figures 1, 2).

**Table 2:** Muscle deficits in the two groups preoperatively and at three, six ,12 and 24 months postoperatively.

Variable	Button (n = 24)	AperFix (n = 24)	P-value
60°/s extensors			
Preoperative	15.93 (10.66-33.97)	16.79 (6.41-35.08)	0.862
3 months	39.01 ± 19.93	45.47 ± 17.87	0.244
6 months	20.4 ± 18.25	23.34 ± 19.78	0.595
12 months	6.2 ± 21.92	11.02 ± 22.35	0.455
24 months	3.69 (-8.66-17.88)	0.76 (-9.84-11.43)	0.959
60°/s flexors			
Preoperative	9.4 ± 16.24	10.88 ± 20.76	0.785
3 months	16.05 ± 16.56	23.84 ± 22.98	0.185
6 months	-3.33 ± 16.85	-1.01 ± 23.88	0.7
12 months	-13.38 ± 26.07	-9.52 ± 24.26	0.598
24 months	-16.35 ± 24.91	-11.16 ± 23.48	0.462
180°/s extensors			
Preoperative	8.57 ± 16.23	12.2 ± 16.47	0.446
3 months	28.98 ± 19.67	37.08 ± 21.09	0.176
6 months	8.43 ± 19.56	11.78 ± 22.72	0.587
12 months	-4.77 ± 20.94	0.54 ± 23.61	0.414
24 months	-7.8 ± 19.91	-7.05 ± 21.24	0.901
180°/s flexors			
Preoperative	5.84 ± 13.05	4.73 ± 16.8	0.801
3 months	11.64 ± 15.95	18.23 ± 18.01	0.187
6 months	-5.19 ± 16.62	-3.54 ± 20.41	0.76
12 months	-14.34 ± 19.27	-6.56 ± 19.57	0.172
24 months	-20.1 ± 25.08	-8.99 ± 19.58	0.094
60°/s H/Q ratio			
Preoperative	-16.99 ± 20.97	-11.23 ± 19.32	0.328
3 months	-38.76 (-74.72-28.07)	-37 (-58.1-14.98)	0.68
6 months	-28.39 (-50.08-11.71)	-28.97 (-49.66-16.75)	0.992
12 months	-24.44 (-32.35-3)	-22.81 (-30.6-13.07)	1
24 months	-19.06 (-28.55-3.24)	-12.31 (-30.34-3.64)	0.613
180°/s H/Q ratio			
Preoperative	-3.13 ± 17.18	-10.51 ± 19.79	0.174
3 months	-30.8 (-49.94-8.61)	-27 (-44.66-7.52)	0.992
6 months	-17.57 (-38.06-2.83)	-17.37 (-38.48-2.4)	0.705
12 months	-4.43 (-28.95-5.39)	-5.23 (-16.15-0)	0.942
24 months	-10.63 (-26.63-1.59)	0 (-12.69-5.42)	0.119

**Figure 3:**
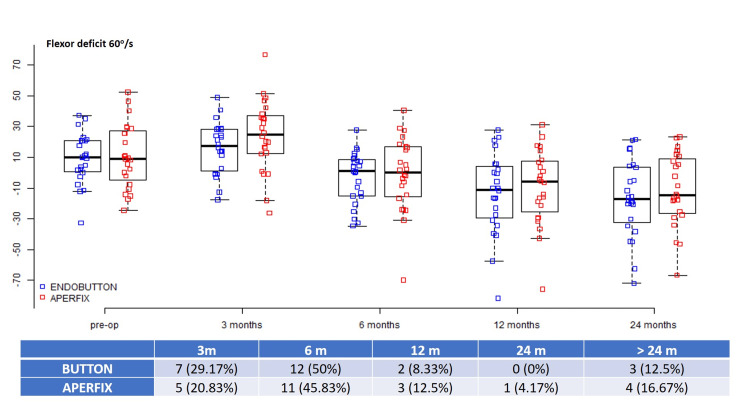
Flexor deficits at 60°/s during different time intervals.

**Figure 4:**
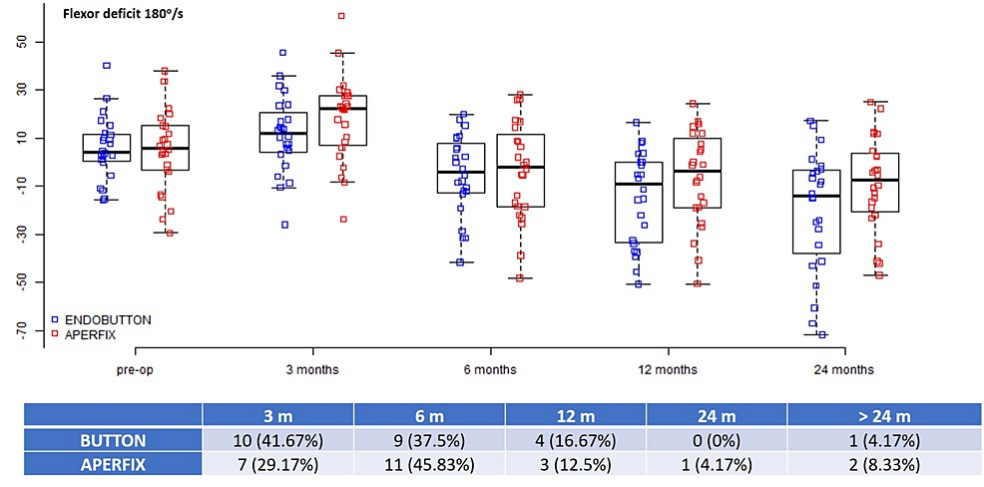
Flexor deficits at 180°/s during different time intervals.

The mean preoperative 60°/s flexor deficit was 9.4 ± 16.24 in the Button group and 10.88 ± 20.76 in the AperFix group (p = 0.875), and the corresponding 180°/s preoperative values were 5.84 ± 13.05 and 4.73 ± 16.8, respectively (p = 0.801) (Table 1). These values were normalized earlier than quadriceps: the 60°/s flexor strength was regained in the first six months in 19 (79.17%) patients in the Button group and in 16 (66.7%) patients in the AperFix group, whereas the percentages for the 180°/s flexor strength were 79.17% for the Button group and 75% for the AperFix group. Beyond the 24-month evaluation, only three (12.5%) patients in the Button group and four (16.67%) patients in the AperFix group had significant 60°/s flexor deficits. Similarly, the 180°/s flexor deficit beyond 24 months was present in one (4.17%) patient in the Button group and two (8.33%) patients in the AperFix group (Figures 3, 4).

Hamstrings/Quadriceps ratio

**Figure 5:**
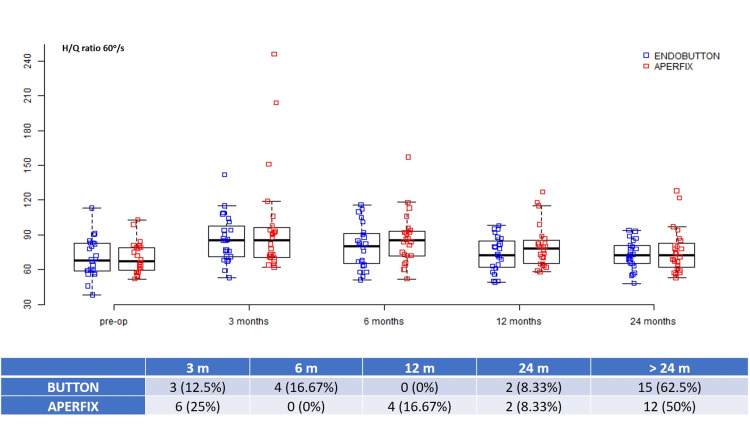
Hamstrings/Quadriceps ratio at 60°/s during different time intervals.

**Figure 6:**
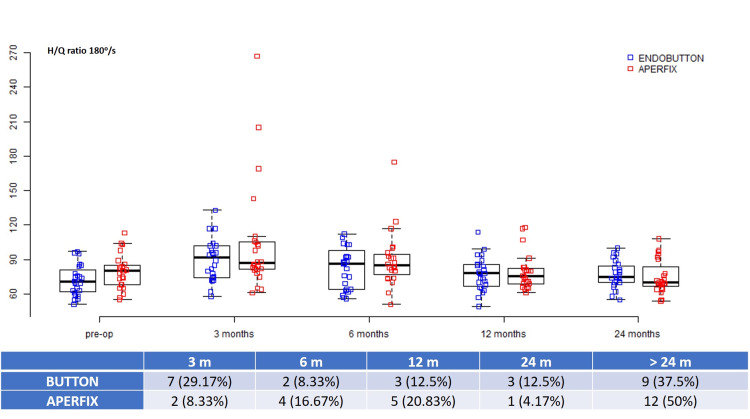
Hamstrings/Quadriceps ratio at 180°/s during different time intervals.

An H/Q ratio of 60-70% for 60°/s and 70-80% for 180°/s was considered safe for RTP in this study. The mean preoperative 60°/s H/Q ratio was -16.99 ± 20.97 for the button group and -11.23 ± 19.32 for the AperFix group (p = 0.328) (Table 2). Accordingly, the 180°/s H/Q ratio was -3.13 ± 17.18 and -10.51 ± 19.79, respectively (p = 0.174). At 60°/s, the mean recovery time was six and 7.5 months for the button and AperFix groups, whereas 15 and 12 patients, respectively, did not recover during the two-year duration. At 180°/s, a mean recovery time of six months was needed for the button group, and nine patients did not recover two years later. For the Aperfix group, nine months were needed, and 12 patients did not recover in two years (Figures 5, 6).

It is obvious that the quadriceps deficits are the most difficult to be restored after an ACL reconstruction as they had the longest recovery time and failure to recover. This is reflected by the H/Q ratio as well as patients also had great difficulty in reaching the H/Q target percentage. This is concerning due to its relevance to a risk of injury.

Functional status

Regarding the secondary outcomes IKDC, Lysholm, Tegner, and KT-1000, no statistically significant differences were found between the AperFix and cortical Button groups at three, six, 12, and 24 months post-surgery (Table 3). Finally, a correlation analysis was performed between the functional tests and muscle deficits. As shown in Table 4, hamstring deficits were correlated with a statistically significant difference in IKDC 2000 and KT-1000 at one year. At two years, the clinical scores had no correlation with muscle deficits.

**Table 3:** Clinical performance and anteroposterior knee stability at different time intervals. IKDC = International Knee Documentation Committee

	IKDC 2000			Tegner scale			Lysholm score			KT-1000 measurement		
	Button	AperFix	P-value	Button	AperFix	P-value	Button	AperFix	P-value	Button	AperFix	P-value
Preoperative	67.88 ± 11.39	61.67 ± 13.24	0.059	3.88 ± 1.26	3.8 ± 1.67	0.466	83.08 ± 10.85	79.32 ± 11/45	0.188	8.71 ± 1.99	9.23 ± 2.48	0.471
3 months	72.41 ± 13.35	68.65 ± 11.5	0.222	4.04 ± 1.23	4.24 ± 1.45	0.621	88.08 ± 9.24	88.21 ± 11.9	0.945	7.27 ± 1.62	8.01 ± 1.48	0.041
6 months	84.39 ± 8.97	82.34 ± 10.22	0.165	5.75 ± 1.33	5.64 ± 1.78	0.678	94.08 ± 6.43	93.56 ± 7.32	0.356	6.35 ± 1.46	6.73 ± 1.07	0.141
12 months	92.33 ± 9.98	91.32 ± 8.34	0.169	7.46 ± 1.56	7.65 ± 1.75	0.988	96.08 ± 5.19	95.89 ± 5.45	0.778	5.79 ± 1.65	6.21 ± 1.11	0.095
24 months	94 ± 11.44	95.32 ± 4.67	0.565	7.83 ± 1.37	8.07 ± 1.54	0.401	97.46 ± 5.03	97.87 ± 3.43	0.566	5.27 ± 1.76	5.63 ± 1.42	0.310

**Table 4:** Correlation of functional scores and anteroposterior knee stability with flexors and extensors muscle deficits. IKDC = International Knee Documentation Committee

	Muscle deficits (rho)			
	Extensors 60°/s	Flexors 60°/s	Extensors 180°/s	Flexors 180°/s
12 months				
IKDC 2000	-21.39% (0.144)	-31.18% (0.031)	-10.88% (0.462)	-31.76% (0.028)
Lyshom	-24.52% (0.093)	-27.58% (0.058)	-10.65% (0.471)	-17.35% (0.238)
KT-1000	27.17% (0.062)	28.39% (0.051)	19.51% (0.184)	35.79% (0.013)
24 months				
IKDC 2000	-12.08% (0.413)	-20.48% (0.163)	-11.68% (0.429)	-28% (0.054)
Lyshom	-13.21% (0.371)	-20.62% (0.16)	-3.17% (0.831)	-22.27% (0.128)
KT-1000	11.78% (0.425)	26.49% (0.069)	21.19% (0.148)	28.49% (0.05)

## Discussion

The main conclusion of our study was that there is no significant statistical difference between expandable and cortical button femoral fixation in ACL reconstruction with hamstring tendons regarding both primary (strength deficits) and secondary (clinical scores and knee stability) outcome measurements. This is in contrast to our hypothesis that the expandable femoral fixation would provide better isokinetic results.

Numerous systematic reviews and meta-analyses have compared the most commonly used femoral fixation methods, including interference screws, cortical buttons (fixed and adjustable), and cross pins, with various studies showing similar results while others demonstrating slight superiority of the interference screw regarding clinical performance and knee stability, as well as more tunnel widening and revision rates for the cortical button fixation [1,2,5-7,15-17]. The AperFix expandable femoral fixation is expected to provide better graft incorporation in a blunt tunnel, more circumferential compression of the graft, less insertional torque compared to interference screws, and the theoretical effect of a double-band ACL equivalent as its design separate (trough graft rotation) the anteromedial and posterolateral bundles of the ACL graft. Recent biomechanical studies of the AperFix system have shown not only better restoration of anterior tibial translation to the intact level at low flexion angles (<30°) compared with button fixation [8] but also strong ultimate failure load and the least amount of cyclic displacement of the graft in the tibial site [18]. For these reasons, we decided to investigate the clinical relevance of these findings compared with our standard practice of cortical button fixation. Regarding the clinical performance of the AperFix system, some studies have reported no significant differences in clinical symptoms, laxity testing, scoring systems, and complication rates [9,10]; however, others, such as the study by Eazazi et al. [11], found that AperFix resulted in better improvement of Lysholm score compared to Rigidfix, and the two performed better than the Endobutton. At the two-year follow-up in this study, there was only one failure in the AperFix group; six cases for the Endobutton group, and 4 cases for the Rigidfix group. In our study, the final clinical outcome and the stability of the knee showed no statistical differences between the different femoral fixation methods in accordance with the existing literature [13].

The main scope of this study was to assess muscle deficits and the readiness to return to sport (RTS) after ACL reconstruction. Although there are several studies on RTP after ACL reconstruction, no consensus has been achieved regarding RTS criteria that can vary from no criteria at all to time-based, subjective, objective, or a combination of all of the above. Earlier studies seem to prefer time-based criteria for RTS with earlier thresholds, usually at the three-months mark for running and six months for cutting/pivoting sports [19,20]. Later studies utilized test batteries for RTS, typically consisting of strength tests, hop tests, and movement quality tests [21,22]. The importance of psychological factors for a safe RTS is also increasingly acknowledged, with self-efficacy, fear of re-injury, and locus of control being predictive of outcomes after ACL reconstruction [23].

One of the most used objective criteria for RTS after ACL reconstruction is quadriceps and hamstrings strength. The Limb Symmetry Index (LSI) is used for this, with different cut-offs depending on the center. Many studies use the 80-90% threshold for LSI in quadriceps and hamstrings isokinetic measurements, especially for recreational groups or simple activities such as running [19,20,23,24]. Other centers are stricter, requiring 100% LSI for return to cutting or pivoting sports [21,22,25]. Our group of patients participated mainly in recreational activities and semi-professional sports activities, except for nine (19%) patients who were at a professional level. As a result, our chosen values for LSI during isokinetic quadriceps and hamstrings assessment at 60°/s and 180°/s was 80%. As indicated by our results, all quadriceps levels reached the aim of 80% LSI in one year, despite our low chosen threshold. Hamstrings, on the other hand, necessitated six months on average to be restored. Even at the one-year interval, however, 19 patients had not reached the target values regarding quadriceps strength, indicating the variance regarding readiness for RTS and the need for a personalized non-time-based decision. On the same note, Capin et al. [26] showed that RTS before nine months yielded a higher chance of ACL reinjury, whereas Grindem et al. [25] showed that RTS after criteria achievement reduced the risk of re-rupture by 75-84%. Losciale et al. [27], on the other hand, claimed that RTS criteria have no impact on second ACL injury rates.

Another strength metric used for RTS is the H/Q ratio at 60°/s and 180°/s, which is known to be correlated with predisposal for re-injury [21,24,28]. Target levels for RTS are 50-80%, with higher levels for faster speeds, leading to our thresholds of 60-70% for 60°/s and 70-80% for 180°/s [23,24]. In our study, the ratio was achieved in a mean time of 6-7.5 months, but the patients failed to maintain this ratio, with 17 patients failing the target goals in two years. This is in agreement with the higher strength deficits at the two-year mark found in our study, especially concerning the quadriceps. The reason behind this could be the lack of follow-through with physiotherapy and exercise regimens; however, considering the higher ACL re-injury rates during the first two years, it should be addressed.

Numerous authors have found a significantly higher ACL incidence when RTS is commanded before six or nine months after surgery [29], whereas Nagelli et al. [28] suggested RTS at two years to fully resolve issues related to ligamentization, bone bruises, proprioception, neuromuscular control, and knee strength. Of high value is a recent meta-analysis by Hurley et al. [30] indicating that RTS criteria significantly reduce ACL graft ruptures, but stricter criteria for RTS do not result in lower ACL re-injury rates. Finally, younger patients are at a higher risk of ACL reinjury after ACL reconstruction and justify the use of a higher threshold for RTS criteria [28].

Strengths and limitations

The main strengths of our study are its prospective randomized design, the inclusion of male patients with similar demographic data and no severe meniscal and/or cartilage pathology, the proper isokinetic evaluation at 60°/s and 180°/s, and the exclusion of patients with additional pathology, small-size grafts, and those who missed the final appointments. However, this study has the following limitations: the small final number of patients, the use of muscle deficits and H/Q ratio as the only criteria for RTS, and the relatively short period (24 ± 8 months) of the final follow-up evaluation.

## Conclusions

Expandable femoral fixation is not superior to the non-adjustable cortical button in terms of stability, objective functional performance, and post-surgery strength deficits. The theoretical advantages of the AperFix system such as strong initial graft fixation, homogenous circular graft compression, no windshield wiper effect, and double band ACL simulation were not proven in our study. In contrast, our study emphasizes the role of RTS criteria and the need for personalized decisions depending on objective data, as even two years later, patients had severe muscle deficits.
